# Perceived fairness of algorithmic decision-making and university teachers’ job burnout: a moderated chain-mediation model

**DOI:** 10.3389/fpsyg.2026.1901039

**Published:** 2026-08-26

**Authors:** Jiwei Wang, Decheng Li, Xin Zhao

**Affiliations:** 1 School of Management, Lanzhou Technology and Business College, Lanzhou, China; 2 Department of Physical Education, Lanzhou University, Lanzhou, China

**Keywords:** algorithm anxiety, job burnout, organizational support, perceived fairness of algorithmic decision-making, psychological safety, university teachers

## Abstract

As big data and artificial intelligence (AI) technologies become embedded in the governance of higher education, algorithmic decision-making has improved managerial efficiency but has also led teachers to question the fairness of evaluation and to face difficulties in psychological adaptation, making job burnout an increasingly salient concern. How perceived fairness of algorithmic decision-making (FAD) relates to university teachers’ job burnout, and through what psychological mechanism, remains unclear. Drawing on the transactional theory of stress and organizational support theory, we built a moderated chain-mediation model in which psychological safety and algorithm anxiety operate as sequential mediators and organizational support acts as a moderator. Questionnaire data were collected from 895 university teachers in Shanghai and Gansu, China, and the model was tested with the SPSS PROCESS macro using standardized coefficients and bias-corrected bootstrapping. Three findings emerged. First, FAD was negatively associated with job burnout, and this relationship was fully accounted for by the proposed mediators. Second, psychological safety and algorithm anxiety each mediated the link and together formed a significant serial path (FAD → psychological safety → algorithm anxiety → job burnout; *β* = −0.002, 95% CI [−0.005, −0.000]); algorithm anxiety was the single strongest mediator, accounting for 23.60% of the total effect. Third, organizational support moderated the algorithm anxiety–burnout association: Johnson–Neyman analysis indicated a significant positive association at low support (below 4.16) that became non-significant in the mid-range and, above 5.36, reversed in sign. Because the design is cross-sectional, this reversal should be read as a boundary pattern that requires replication rather than as evidence of a causal switch. By situating FAD, psychological safety, algorithm anxiety, and job burnout within one framework, the study clarifies the technology–individual–organization interplay and offers evidence for fairer algorithmic governance and for protecting teachers’ occupational health.

## 1 Introduction

Big data and artificial intelligence are working their way deep into how universities are run. Intelligent evaluation systems now shape the management of teaching and research along efficient, quantifiable lines, lending fresh technological support to evaluation-driven education (Kellogg et al., 2020; Li et al., 2025). Those efficiency gains come with a cost, however: a widening set of fairness disputes and a harder problem of psychological adjustment. When algorithmic decisions strike people as opaque or unfair, members of an organization slide easily into anxiety, distrust, and a sense of estrangement, and their work engagement and professional identity suffer for it (Lee, 2018). University teachers feel this strain acutely. Their work is professional, creative, and tied to context in ways that numbers cannot fully register, so an algorithm that leans on measurable data and overlooks qualitative contributions chips away at their autonomy and sense of meaning, and the risk of burnout climbs with it (Watts and Robertson, 2011; Sabagh et al., 2018; Zou, 2023).

Burnout is the exhaustion—emotional exhaustion, depersonalization, and a shrinking sense of accomplishment—that settles in under prolonged work stress. Among university teachers it has kept rising, pushed not only by familiar pressures such as performance accountability and up-or-out tenure but also by the upheavals of digital change (Sabagh et al., 2018; Zhang et al., 2025). Seen through a person–environment lens, burnout grows out of a lasting mismatch between the teacher and the work setting (Watts and Robertson, 2011). Algorithmic governance can deepen that mismatch: when teachers sense that algorithms are taking over their professional judgment and that the decisions behind them are opaque and possibly biased, the resulting estrangement is amplified and the risk of burnout grows (Kellogg et al., 2020). Fairness in algorithmic decision-making, then, is no longer a purely technical matter; it reaches directly into teachers’ occupational health.

What stays unclear is the psychological route from FAD to burnout. We propose that psychological safety and algorithm anxiety are the pivotal links. Psychological safety is a teacher’s trust that speaking honestly, owning a mistake, or asking for help will not bring punishment (Edmondson, 1999); when algorithmic decisions lack fairness and transparency, the work environment feels less certain and that safety erodes. Algorithm anxiety is the worry and resistance that algorithmic decisions provoke—over their opacity, their handling of privacy, their potential bias, and the loss of control they imply (Zha et al., 2022)—and it steadily drains psychological resources while feeding burnout (Zhan and Chen, 2025). The two are ordered rather than separate: stronger psychological safety cushions uncertainty and holds anxiety in check, which points to a chain running from perceived fairness through psychological safety and algorithm anxiety to job burnout.

Building on this, we draw together the transactional theory of stress (Lazarus and Folkman, 1984) and organizational support theory (Eisenberger et al., 1986) and, with a sample of 895 university teachers, build and test a moderated chain-mediation model. The model traces the serial mediating roles of psychological safety and algorithm anxiety between FAD and burnout and asks whether organizational support conditions the final link from algorithm anxiety to burnout, so as to sketch a technology–individual–organization account of the process. The contribution is threefold. To our knowledge, this is the first study to bring FAD, psychological safety, algorithm anxiety, and job burnout into one framework, joining two strands of work—algorithmic governance and teachers’ occupational health—that rarely meet. It also traces the psychological route from FAD to burnout, and in particular the serial mediation through psychological safety and algorithm anxiety. By treating organizational support as a boundary condition, it further shows how organizational resources shape the passage from anxiety to burnout, offering lessons for a people-centered approach to intelligent governance in education and for the design of targeted interventions.

## 2 Theoretical background and literature review

### 2.1 Perceived fairness of algorithmic decision-making (FAD)

Perceived fairness of algorithmic decision-making captures how fair a person judges the procedures and outcomes of an algorithmic system to be (Starke et al., 2022). The construct inherits the familiar three-part structure of organizational justice (Cropanzano and Ambrose, 2015; Colquitt et al., 2001). Translated to the algorithmic setting, distributive justice concerns whether algorithmic outputs are reasonable, procedural justice concerns whether the decision rules are consistent and transparent, and interactional justice concerns whether the system leaves room for individual needs and appeals (Lee, 2018; Cropanzano and Ambrose, 2015). Lee (2018) showed that people accept algorithmic decisions largely to the extent that they see them as fair, and that algorithms are judged unfair when they look rigid and blind to individual circumstances. A systematic review reached a similar conclusion, naming outcome accuracy and process transparency as the main drivers of fairness perceptions (Starke et al., 2022). Acceptance is not uniform, though. Depending on the task, people sometimes shun algorithmic judgment and sometimes prefer it, a contrast captured by the literatures on algorithm aversion and algorithm appreciation (Dietvorst et al., 2015; Logg et al., 2019).

Fairness perceptions take on a particular character in higher education. Teaching evaluation and judgments about academic innovation are subjective tasks: they lean on the evaluator’s contextual judgment far more than objective tasks such as allocating resources or scheduling courses. Turn an algorithm to these subjective tasks without room for professional judgment, and teachers’ sense of fairness and their trust both fall sharply (Castelo et al., 2019; Longoni et al., 2019; Wang and Zhang, 2023). In teacher evaluation the construct surfaces on three fronts at once: whether the result reflects the full range of a teacher’s work (outcome fairness), whether the standards are transparent and applied consistently (procedural fairness), and whether teachers can take part, appeal, and be heard (interactional fairness). Let any one of these slip, and overall trust in the system erodes while resistance grows (Lee, 2018; Cropanzano and Ambrose, 2015).

### 2.2 Job burnout

Job burnout is the exhaustion that builds up when a person can no longer cope with prolonged work stress. Maslach’s three-factor model remains the dominant lens, treating burnout as the joint presence of emotional exhaustion, depersonalization, and reduced personal accomplishment (Maslach and Leiter, 1997; Maslach et al., 2001). Emotional exhaustion, the fatigue that follows from drained emotional reserves, is usually taken as the core of the syndrome. Depersonalization shows up as detached, indifferent, or cynical attitudes toward work, while reduced personal accomplishment appears as a habit of judging one’s own contribution harshly.

University teachers sit squarely in the high-risk category. As higher education has moved into its mass phase, they have shouldered heavier performance accountability, the survival logic of up-or-out systems, and the constant pull between teaching and research; declining enthusiasm, physical and mental fatigue, and a creeping sense of mediocrity are common results (Watts and Robertson, 2011). Surveys repeatedly find burnout of varying severity among faculty, traceable to a mix of personal traits, job demands, and organizational resources (Sabagh et al., 2018). What this body of work has largely passed over is digital technological change, and algorithmic management in particular, as a new source of strain (Kellogg et al., 2020).

### 2.3 Psychological safety

Psychological safety is the belief that one can take interpersonal risks at work, such as voicing dissent, admitting a mistake, or asking for help, without being penalized for it (Edmondson, 1999). Edmondson (1999) showed that trust among colleagues shapes performance through this sense of safety, and that where it is high people fear failure less and are readier to try new things (Edmondson and Lei, 2014). The feeling grows out of a broader climate of trust, reflecting how a person reads their leaders, their colleagues, and the organization as a whole (Mayer et al., 1995).

Algorithmic governance makes psychological safety especially salient. Once algorithms enter management, opaque decision processes, fuzzy evaluation standards, and outcomes that resist explanation all sharpen teachers’ sense of uncertainty about their work environment. Psychological safety weakens in turn, and defensive responses follow, from reduced engagement to keeping one’s real views to oneself (Edmondson, 1999). What is easy to miss is that psychological safety does not rest on the fairness of the algorithm alone. It also depends on whether the organization backs its teachers and tolerates honest error; perceived organizational support strengthens trust and belonging and is itself an important antecedent of psychological safety (Kurtessis et al., 2017; Frazier et al., 2017).

### 2.4 Algorithm anxiety

Algorithm anxiety is the worry, unease, and resistance people feel when they face algorithmic decision systems (de Vries and Schinkel, 2019). At root it is a specific form of technostress, provoked here by algorithmic decision-making (Ragu-Nathan et al., 2008). Zha et al. (2022) tease apart four strands: cognitive anxiety about the algorithmic black box, fairness anxiety about bias, privacy anxiety about the exposure of personal information, and autonomy anxiety about losing control. The unease has two sources, namely not grasping how the algorithm works and bracing for what it might do (Yao et al., 2026).

How anxious a teacher becomes is bound up with their algorithmic literacy. Those who lack the knowledge and skill to make sense of algorithms are the most prone to anxiety (Zha et al., 2022); where teachers can instead exert some agency over the system, a measure of control returns and the anxiety eases (Kellogg et al., 2020; Jiang and Zhong, 2022). In universities the deeper worry is about professional autonomy. When evaluation leans too hard on algorithms and sidelines professional judgment, feelings of powerlessness and anxiety grow (Yao et al., 2026). Algorithm-failure anxiety, the fear that the system will err or break down, matters most of all: teachers who see algorithms as opaque, unreliable, or biased report strong failure anxiety, which lowers their trust in the organization and raises their risk of burnout (Yao et al., 2026).

### 2.5 Organizational support

Organizational support is an employee’s overall sense that the organization values their contribution, cares about their welfare, and will help when needed (Eisenberger et al., 1986). For university teachers it runs from teaching resources to psychological care to technical training, and it bears directly on their work attitudes and states of mind (Rhoades and Eisenberger, 2002; Xiong and Luo, 2008). When support is adequate it can make up for shortfalls in a teacher’s own psychological resources and hold burnout in check (Rhoades and Eisenberger, 2002; Kurtessis et al., 2017); by deepening belonging and trust, it also lays groundwork for psychological safety (Kahn, 1990).

### 2.6 An integrated theoretical framework

This study draws the transactional theory of stress, the job demands–resources (JD-R) model, and organizational support theory into a single account of how FAD relates to university teachers’ job burnout.

The transactional theory of stress sets out the basic sequence (Lazarus and Folkman, 1984). Faced with a possible stressor, a person first makes a primary appraisal of whether it is threatening, then a secondary appraisal of the resources and control they can bring to bear, and reacts emotionally on that basis. In the algorithmic setting, FAD anchors the primary appraisal, since an unfair algorithm reads as a threat; psychological safety stands in for the secondary appraisal of how controllable the organizational environment feels and what it offers. When the threat looms large and resources fall short, algorithm anxiety is the emotional response that follows, and through the depletion of resources it is tied to burnout. This progression—from a primary appraisal of fairness, to a secondary appraisal of safety, to the emotional response of anxiety, to the depletion that ends in burnout—is what the chain-mediation path in this study is built to capture.

The JD-R model fills in how resources are drained and replenished (Bakker and Demerouti, 2017; Demerouti et al., 2001). In its terms, unfair algorithmic evaluation is a job demand that keeps eating into psychological resources, while psychological safety and organizational support count as job resources; burnout risk rises when demands outrun resources and falls when resources are ample enough to absorb them. Organizational support theory adds that employees who feel cared for and recognized by their organization hold an important reserve for withstanding stress and protecting their mental health (Eisenberger et al., 1986). On this reasoning, organizational support should condition how strongly algorithm anxiety carries through to burnout: where support is plentiful, teachers have more to draw on in coping with anxiety, and its push toward burnout softens.

**Figure 1:**
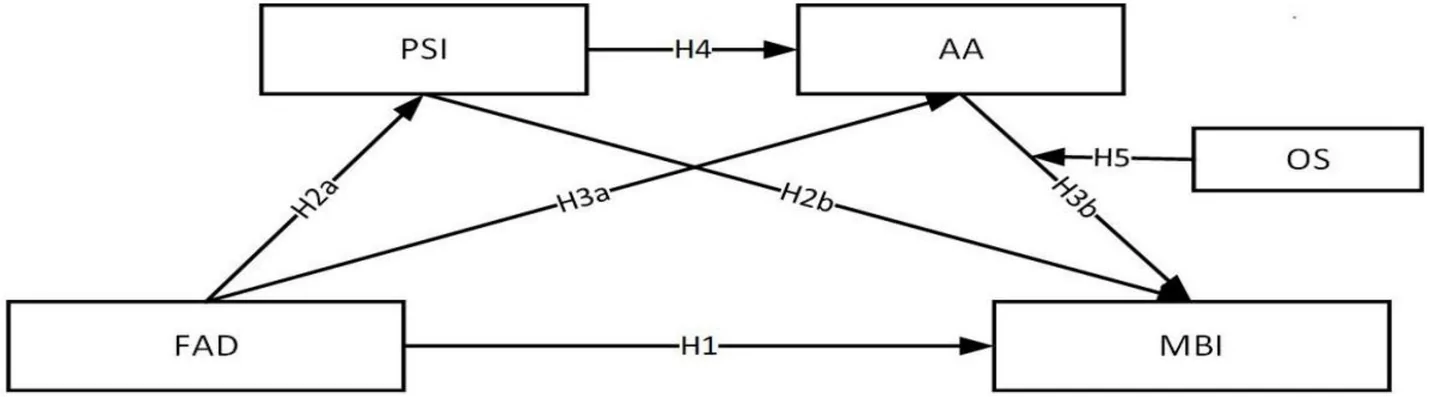
The hypothesized moderated chain-mediation model. Perceived fairness of algorithmic decision-making (FAD) is proposed to relate to job burnout serially through psychological safety and then algorithm anxiety (H1–H4); organizational support is modeled as a moderator of the algorithm anxiety → job burnout path (H5). Solid arrows denote the hypothesized direct and mediating paths; the arrow from organizational support denotes the moderating effect. Gender, age, professional title, discipline, teaching experience, and weekly working hours were included as covariates.

The study advances a moderated chain-mediation model (Figure 1) in which FAD is linked to teachers’ job burnout by way of psychological safety and then algorithm anxiety, with organizational support conditioning the later stretch of that path. By pairing the moving parts of stress appraisal with the depletion-and-buffering logic of job resources, the framework offers one coherent way of reading teachers’ occupational health in the algorithmic era.

## 3 Hypothesis development

### 3.1 FAD and job burnout

In the terms of the transactional theory of stress, an unfair algorithmic decision is a job demand that primary appraisal flags as a threat (Lazarus and Folkman, 1984). Teachers who feel that algorithmic evaluation misses the substance of their teaching, research, and professional growth come away with a strong sense of unfairness and lost control, and their enthusiasm drains as negative attitudes set in (Lee, 2018). The association has empirical backing. Algorithms seen as unfair erode user trust and can tip into outright aversion (Lee, 2018; Dietvorst et al., 2015; Wang et al., 2023), and unfair treatment at work, such as microaggressions, tracks positively with burnout and ranks among its notable risk factors (Ogunnowo et al., 2024). Inside the university, an algorithm that fixates on countable indicators while passing over qualitative contributions leaves teachers feeling that their professional worth is discounted, which feeds emotional exhaustion and depersonalization (Sabagh et al., 2018). We therefore propose:

*H1*. Perceived fairness of algorithmic decision-making negatively predicts university teachers’ job burnout.

### 3.2 The mediating role of psychological safety

Fair, transparent algorithmic decisions give teachers a firmer sense that their work environment is predictable and within their control, and psychological safety rises with it; opaque, unfair evaluation does the reverse, feeding uncertainty and chipping away at that safety (Edmondson, 1999). People who trust that leaders and colleagues will hear them out tend to work more openly and with more commitment (Dirks and Ferrin, 2002). Psychological safety is one of the resources that keep burnout at bay: low safety is a signature of badly burned-out teachers and helps carry the effect of stressors onto burnout, with strains like work pressure and employee silence raising burnout precisely by wearing safety down (Kassandrinou et al., 2023). More recent work points the same way, linking teachers’ appraisals of emerging technologies such as AI to their burnout through psychological channels (Wang and Zhou, 2025) and reporting a steady negative association between psychological safety and burnout (Qi and Yu, 2021). This pattern suggests that FAD reaches burnout partly by an indirect route, through psychological safety. We therefore propose:

*H2*. Psychological safety mediates the relationship between FAD and job burnout; specifically, FAD is positively associated with psychological safety (H2a), and psychological safety is negatively associated with job burnout (H2b).

### 3.3 The mediating role of algorithm anxiety

Cognitive appraisal theory holds that an algorithm appraised as a threat sets off negative emotion (Lazarus and Folkman, 1984). Facing algorithmic evaluation, teachers grow anxious over the black box, the prospect of bias, privacy exposure, and the thinning of their autonomy (Zha et al., 2022); the less they understand and can manage the system, the sharper that helplessness and anxiety become (Zhan and Chen, 2025). Sustained algorithm anxiety keeps draining psychological resources and pulling down engagement, which worsens emotional exhaustion and depersonalization (Yao et al., 2026). There is also evidence that algorithm-failure anxiety mediates the link between how users perceive an algorithm and what they intend to do (Yao et al., 2026). FAD, then, may reach burnout indirectly through algorithm anxiety as well. We therefore propose:

*H3*. Algorithm anxiety mediates the relationship between FAD and job burnout; specifically, FAD is negatively associated with algorithm anxiety (H3a), and algorithm anxiety is positively associated with job burnout (H3b).

### 3.4 The chain-mediating role of psychological safety and algorithm anxiety

The two mediators are not parallel but ordered. In appraisal terms, psychological safety is an outcome of secondary appraisal and sets the gain on a teacher’s emotional reaction to algorithmic threat. A teacher who feels safe believes they have the latitude and resources to handle algorithmic uncertainty, which keeps anxiety from taking hold; a teacher who does not feel safe reads the algorithm as more threatening, and anxiety climbs (Edmondson, 1999; Zhan and Chen, 2025). That anxiety then drains psychological resources further, is reciprocally tied to still-lower safety, and ends in burnout.

Recent studies offer both direct and parallel support for this sequence. Psychological safety correlates negatively with burnout (Qi and Yu, 2021); intolerance of uncertainty, a close relative of algorithm anxiety, predicts higher burnout (Gao et al., 2025); and serial-mediation designs have captured how burnout forms, with an antecedent reaching burnout through two ordered mediators such as stress and insomnia, or resilience and change fatigue (Gao et al., 2025; Wang et al., 2024). We therefore propose:

*H4*. Psychological safety and algorithm anxiety serve as chain mediators between FAD and job burnout; that is, higher FAD is associated with greater psychological safety, lower algorithm anxiety, and, in turn, lower job burnout.

### 3.5 The moderating role of organizational support

Both the JD-R model and organizational support theory hold that job resources cushion the way job demands wear people down toward burnout (Eisenberger et al., 1986; Bakker and Demerouti, 2017). Algorithm anxiety is a new kind of technological demand, and how strongly it carries through to burnout should depend on the resources a teacher can draw on. Organizational support is one such resource, supplying emotional reassurance, informational guidance, and institutional backing. Where support runs high, teachers are better placed to turn algorithm anxiety into constructive coping, and its push toward burnout weakens; where support is thin, nothing absorbs the anxiety and its positive association with burnout shows through in full (Xu and Yang, 2021). Other work agrees that organizational support and related resources moderate the stress–burnout relationship and can interrupt the passage from stress to burnout (Jianwu and Xiangqian, 2013), and evidence that more transparent, explainable algorithms can rebuild trust and ease anxiety when results disappoint points the same way (Kizilcec, 2016). We therefore propose:

*H5*. Organizational support moderates the effect of algorithm anxiety on job burnout; under high organizational support, the positive effect of algorithm anxiety on job burnout is significantly attenuated.

## 4 Materials and methods

### 4.1 Participants

This study used random sampling to administer electronic questionnaires to university teachers in Shanghai and Gansu, China. A total of 923 questionnaires were returned, and after excluding invalid responses, 895 valid questionnaires were obtained, yielding an effective response rate of 96.97%. The sample was dominated by women (57.88%), teachers aged 36 and above (73.29%), and those holding associate-senior titles (50.95%); the disciplinary distribution covered the humanities and social sciences (34.86%), science/engineering/agriculture/medicine (31.84%), and education (27.82%), among others; teaching experience was mostly concentrated within 10 years (76.65%), and work intensity was generally high, with 59.00% working more than 41 h per week. The sample structure broadly corresponds to the overall distribution of university teachers in China; details are presented in Table 1.

**Table 1:** Frequency analysis of demographic characteristics.

Variable	Category	Frequency	Percentage (%)
Gender	Male	377	42.12
	Female	518	57.88
Age	Under 25	8	0.89
	26–35	231	25.81
	36–45	401	44.80
	Over 46	255	28.49
Professional title	Full senior	148	16.54
	Associate senior	456	50.95
	Intermediate	237	26.48
	Junior or below	54	6.03
Discipline	Humanities and social sciences	312	34.86
	Sci./eng./agri./medicine	285	31.84
	Education	249	27.82
	Other	49	5.47
Teaching experience (years)	0–5	309	34.53
	6–10	377	42.12
	11–15	161	17.99
	Over 16	48	5.36
Weekly working hours	Under 20	43	4.80
	21–40	324	36.20
	41–60	328	36.65
	Over 61	200	22.35

Shanghai and Gansu were chosen deliberately to span the range of higher-education digitalization in China. Shanghai is an economically advanced eastern municipality whose universities were among the first to adopt data-driven and algorithmic tools in teaching evaluation, performance appraisal, and promotion review. Gansu, by contrast, is a less-developed western province in which such systems have been introduced more slowly and unevenly. Recruiting teachers from these two settings widens the variation in algorithmic exposure, organizational resources, and fairness perceptions, and thus subjects the hypothesized relationships to a more demanding test than a single-region sample would allow. We return to the limits of this two-province design, and to the institutional and regional differences that may shape generalizability, in the Limitations section.

### 4.2 Measures

The questionnaire comprised demographic information and scales for five core variables. All items were rated on a 7-point Likert scale (1 = strongly disagree, 7 = strongly agree), with higher scores indicating higher levels of the corresponding construct. Perceived fairness of algorithmic decision-making was adapted from Colquitt’s (2001) organizational justice scale, covering distributive, procedural, and interactional justice (9 items). Job burnout was measured using the Maslach Burnout Inventory (MBI; Maslach and Leiter, 1997), comprising emotional exhaustion, depersonalization, and reduced personal accomplishment. Psychological safety was assessed by integrating Edmondson’s (1999) team psychological safety scale and May et al.’s (2004) individual psychological safety scale (6 items). Algorithm anxiety was adapted from the algorithm anxiety scale of Zhan and Chen (2025) (5 items). Organizational support was based on Eisenberger et al. (1986), covering organizational care, resource provision, and organizational commitment. Reliability and confirmatory factor analyzes showed that the Cronbach’s α coefficients of the scales ranged from 0.821 to 0.949, and the fit indices (NFI ≥ 0.986, GFI ≥ 0.988, AGFI ≥ 0.979, RMSEA ≤ 0.029) all met measurement standards, indicating good reliability and validity (Table 2).

**Table 2:** Reliability and model-fit indices.

No.	Scale	Cronbach’s α	NFI, GFI, AGFI, RMSEA
1	Perceived fairness of algorithmic decision-making scale	0.821	0.993, 0.994, 0.989, 0.003
2	Maslach Burnout Inventory (MBI)	0.894	0.997, 0.995, 0.991, 0.0001
3	Psychological safety scale	0.942	0.998, 0.997, 0.992, 0.010
4	Algorithm anxiety scale	0.949	1.000, 0.999, 0.998, 0.0001
5	Organizational support scale	0.840	0.986, 0.988, 0.979, 0.029

All five scales are established instruments with documented reliability and validity in prior research. The algorithm anxiety scale (Zhan and Chen, 2025) was originally developed in Chinese and was used directly. The remaining measures, which originated in English (perceived fairness, job burnout, psychological safety, and organizational support), were translated into Chinese by the bilingual research team and checked against the source items to ensure conceptual equivalence; item wording was then adapted to the higher-education algorithmic-evaluation context, framing the items around teaching, research, and promotion evaluation rather than generic management settings. No separate pilot study was conducted. Instead, the psychometric adequacy of the measures in the present sample was evaluated directly: confirmatory factor analysis was carried out for each scale, and as reported in Table 2 the fit indices (NFI, GFI, AGFI, and RMSEA) all met conventional thresholds, which, together with the Cronbach’s α coefficients, supports the reliability and factorial validity of the measures.

## 5 Results

### 5.1 Common method bias

Two procedures were used to assess common method bias (Podsakoff et al., 2003). First, Harman’s single-factor test was applied: an unrotated exploratory factor analysis extracted 15 factors with eigenvalues greater than 1, and the first factor explained only 16.346% of the variance, well below the 40% threshold. Second, a single-common-factor confirmatory model was estimated in which all 39 items of the five scales were constrained to load on one latent factor. This model fit the data very poorly (*χ*^2^/*df* = 24.33, CFI = 0.284, TLI = 0.244, RMSEA = 0.162 [90% CI 0.144, 0.169], SRMR = 0.167), showing that a single method factor cannot reproduce the observed covariance structure. These results indicate that common method bias is unlikely to account for the findings.

### 5.2 Descriptive statistics and correlations

Descriptive statistics and correlations among the variables are presented in Table 3. FAD was significantly and negatively correlated with job burnout (*r* = −0.375, *p* < 0.01) and algorithm anxiety (*r* = −0.515, *p* < 0.01), and significantly and positively correlated with psychological safety (*r* = 0.201, *p* < 0.01); psychological safety was significantly and negatively correlated with job burnout (*r* = −0.225, *p* < 0.01) and algorithm anxiety (*r* = −0.291, *p* < 0.01); and algorithm anxiety was significantly and positively correlated with job burnout (*r* = 0.292, *p* < 0.01). The directions of these correlations are all consistent with the hypotheses. Drawing on prior research and accounting for the potential confounding of demographic factors, gender, age, professional title, discipline, teaching experience, and weekly working hours were included as control variables in the subsequent models.

**Table 3:** Descriptive statistics and correlations.

Variable	*M*	SD	1	2	3	4
1 FAD	3.932	1.271	1			
2 MBI	3.446	1.418	−0.375^**^	1		
3 PSI	4.208	1.687	0.201^**^	−0.225^**^	1	
4 AA	4.152	1.813	−0.515^**^	0.292^**^	−0.291^**^	1

FAD, perceived fairness of algorithmic decision-making; MBI, job burnout; PSI, psychological safety; AA, algorithm anxiety. Values are zero-order correlations. ^**^p < 0.01.

### 5.3 Test of the chain-mediation model

After standardizing all variables, the chain-mediation effect was tested using Model 6 of the SPSS PROCESS macro, with the aforementioned demographic variables as controls (Hayes, 2018). The regression results (Table 4) show that FAD positively predicted psychological safety (*β* = 0.298, *t* = 2.664, *p* < 0.01) and negatively predicted algorithm anxiety (*β* = −0.392, *t* = −6.275, *p* < 0.001); psychological safety negatively predicted algorithm anxiety (*β* = −0.110, *t* = −5.896, *p* < 0.001) and job burnout (*β* = −0.041, *t* = −2.506, *p* < 0.05); and algorithm anxiety positively predicted job burnout (*β* = 0.063, *t* = 2.204, *p* < 0.05).

**Table 4:** Regression analysis of the variables in the model.

Outcome	Predictor	*R* ^2^	*F*	*β*	SE	*t*	LLCI	ULCI
PSI	Constant	0.0734	10.0347^***^	2.6502	0.8704	3.0447^**^	0.9418	4.3586
	FAD			0.2980	0.1119	2.6638^**^	0.0784	0.5176
AA	Constant	0.7522	336.1612^***^	2.2160	0.4866	4.5545^***^	1.2611	3.1709
	FAD			−0.3919	0.0625	−6.2751^***^	−0.5145	−0.2694
	PSI			−0.1101	0.0187	−5.8962^***^	−0.1467	−0.0734
MBI	Constant	0.8397	235.19^***^	6.0681	0.4200	14.4493^***^	5.2438	6.8923
	FAD			−0.0662	0.0545	−1.2149	−0.1731	0.0407
	PSI			−0.0407	0.0162	−2.5064^*^	−0.0726	−0.0088
	AA			0.0632	0.0287	2.2035^*^	0.0069	0.1194

Gender, age, professional title, discipline, teaching experience, and weekly working hours were controlled but are omitted from the table for brevity. ^*^p < 0.05, ^**^p < 0.01, ^***^p < 0.001.

**Figure 2:**
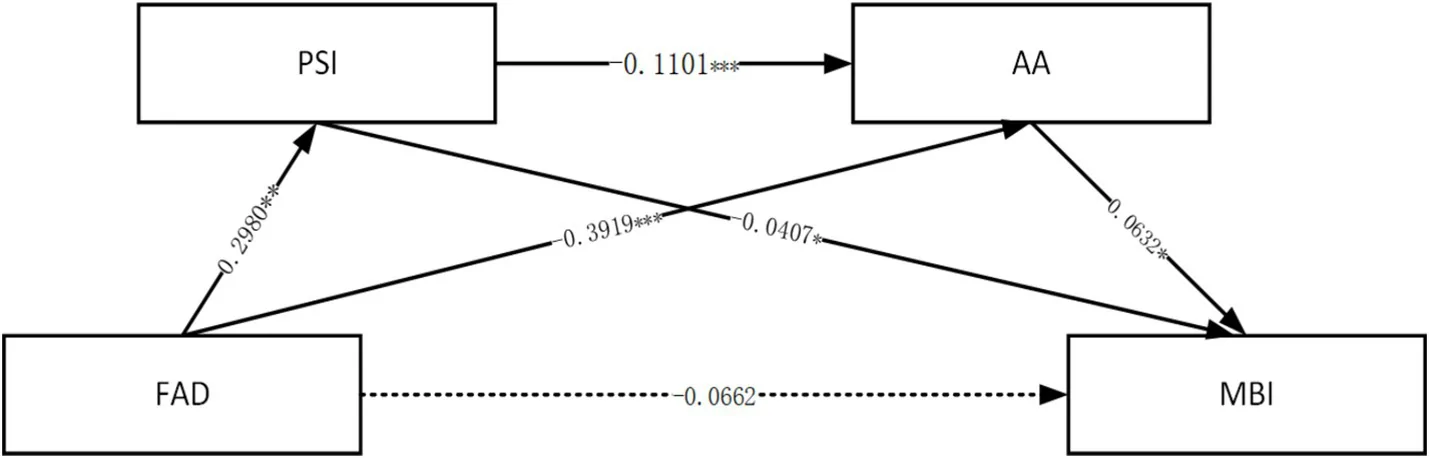
The chain-mediation model with standardized path coefficients.

Bias-corrected bootstrap tests with 5,000 resamples (Preacher and Hayes, 2008) were used to probe the indirect effects (Table 5). The total effect of FAD on job burnout was significant, whereas the direct effect was not, indicating full mediation. All three indirect paths were significant: psychological safety alone accounted for 31.03% of the total indirect effect (supporting H2), algorithm anxiety alone for 63.59% (supporting H3), and the serial path through psychological safety and then algorithm anxiety was also significant (supporting H4). The corresponding coefficients and 95% confidence intervals are reported in Table 5 (Figure 2).

**Table 5:** Results of the chain-mediation effect test.

Effect	Path	Effect	SE	LLCI	ULCI
Direct effect	FAD ⇒ MBI	−0.0662	0.0545	−0.1731	0.0407
Indirect effect	Total indirect effect	−0.0390	0.0146	−0.0691	−0.0121
	FAD ⇒ PSI ⇒ MBI	−0.0121	0.0066	−0.0271	−0.0015
	FAD ⇒ AA ⇒ MBI	−0.0248	0.0125	−0.0505	−0.0020
	FAD ⇒ PSI ⇒ AA ⇒ MBI	−0.0021	0.0014	−0.0053	−0.0001
Total effect	FAD ⇒ MBI	−0.1051	0.0534	−0.2100	−0.0003

### 5.4 Test of the moderation effect

Organizational support was modeled as a moderator using Model 87 of the PROCESS macro. Among Hayes’s templates, Model 87 is the one that combines a two-mediator serial (chain) mediation with a moderator acting on the path from the second mediator to the outcome; it therefore maps directly onto the hypothesized structure, in which FAD is transmitted to burnout serially through psychological safety and then algorithm anxiety (H4) and organizational support conditions the final algorithm anxiety → job burnout link (H5). As shown in Table 6, the algorithm anxiety × organizational support interaction significantly and negatively predicted job burnout (*β* = −0.096, *t* = −8.283, *p* < 0.001), indicating that organizational support moderates the algorithm anxiety–burnout association.

**Table 6:** Chain-mediation results under the moderation of organizational support.

Outcome	Predictor	*R* ^2^	*F*	*β*	SE	*t*	LLCI	ULCI
PSI	Constant	0.0734	10.0347	2.6502	0.8704	3.0447^**^	0.9418	4.3586
	FAD			0.2980	0.1119	2.6638^**^	0.0784	0.5176
AA	Constant	0.7522	336.1612	2.2160	0.4866	4.5545^***^	1.2611	3.1709
	FAD			−0.3919	0.0625	−6.2751^***^	−0.5145	−0.2694
	PSI			−0.1101	0.0187	−5.8962^***^	−0.1467	−0.0734
MBI	Constant	0.7319	219.182	4.5210	0.4700	9.628^***^	3.5990	5.4430
	FAD			−0.0630	0.0520	−1.2160	−0.1650	0.0390
	PSI			−0.0410	0.0160	−2.652^**^	−0.0720	−0.0110
	AA			0.4540	0.0550	8.267^***^	0.3460	0.5620
	OS			0.3080	0.0520	5.968^***^	0.2070	0.4100
	AA×OS			−0.0960	0.0120	−8.283^***^	−0.1190	−0.0730

OS, organizational support. ^**^p < 0.01, ^***^p < 0.001.

**Figure 3:**
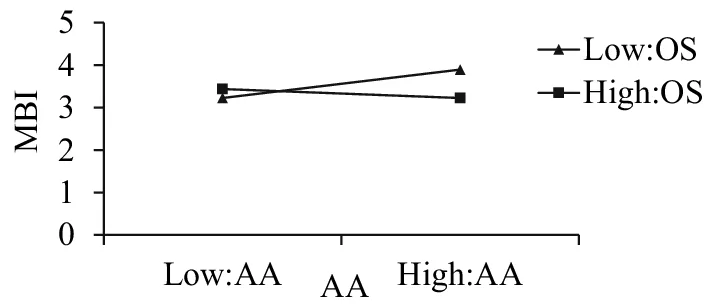
The moderating effect of organizational support on the algorithm anxiety–burnout relationship.

**Figure 4:**
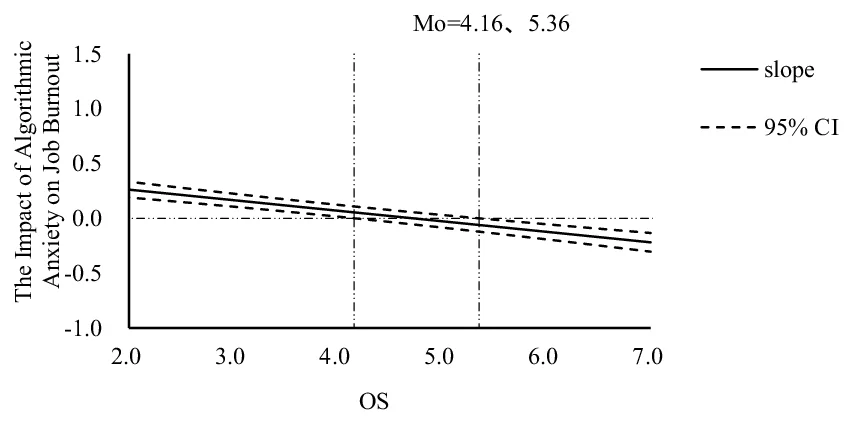
Johnson–Neyman plot for the moderating effect of organizational support.

Simple-slope analysis showed that at low organizational support, algorithm anxiety was significantly and positively associated with job burnout (*β* = 0.184, *p* < 0.001), and this slope flattened as support increased. The Johnson–Neyman procedure located three regions: below an organizational-support value of 4.16 the association was significantly positive (e.g., OS = 1.20, *β* = 0.339, *p* < 0.001); between 4.16 and 5.36 it was non-significant; and above 5.36 it became significantly negative (e.g., OS = 7.00, *β* = −0.219, *p* < 0.001). These patterns support H5. We interpret the sign reversal at high support cautiously: it shows that organizational support buffers, and at high levels offsets, the anxiety–burnout association in this cross-sectional sample, but whether anxiety becomes genuinely protective is a question for future longitudinal and experimental work (Figures 3, 4).

## 6 Discussion

This study constructed and tested a moderated chain-mediation model, revealing the psychological mechanism and boundary conditions through which FAD affects university teachers’ job burnout.

### 6.1 The core role of FAD

FAD showed a significant negative total association with job burnout, and this association was fully carried by psychological safety and algorithm anxiety (H1 supported). The pattern is consistent with Lee’s (2018) work on how algorithmic fairness shapes user trust and emotional responses, and with the task-objectivity account: when algorithms are applied to inherently subjective work such as teaching and research, and individual context and professional judgment are not respected, teachers report a stronger sense of unfairness, a weaker sense of meaning, and more emotional exhaustion (Castelo et al., 2019). Because teaching and research are creative and only partly quantifiable, teachers appear especially sensitive to the rigid logic of algorithmic metrics; when measurable indicators crowd out qualitative contributions, the resulting devaluation of professional worth echoes long-standing arguments that algorithmic management erodes labor autonomy (Kellogg et al., 2020; Li and Bu, 2023). Algorithmic fairness is in this sense less a narrow technical property than an institutional condition tied to teachers’ professional dignity—a point we develop into concrete governance proposals in Section 7.

### 6.2 The mediating and chain mechanisms of psychological safety and algorithm anxiety

Psychological safety and algorithm anxiety each mediated the FAD–burnout association and, in sequence, formed a significant chain (H2, H3, and H4 supported). Algorithm anxiety was the dominant mediator, accounting for 63.59% of the total indirect effect and 23.60% of the total effect, whereas psychological safety played a smaller but reliable role. The ordering of the two mediators is theoretically informative. A fairer algorithmic environment is associated with greater psychological safety, which is in turn associated with lower algorithm anxiety and, finally, with lower burnout—a sequence that maps onto the primary-appraisal → secondary-appraisal → emotional-response logic of the transactional theory of stress (Lazarus and Folkman, 1984) and extends Edmondson’s (1999) account of psychological safety from team learning to algorithmic governance. One practical reading follows: raising algorithmic transparency on its own may not be enough, because without a foundation of psychological safety the same algorithmic information can be appraised through a more threatening lens, leaving the anxiety pathway intact (Sabagh et al., 2018). The deeper source of teachers’ algorithm anxiety appears to be an erosion of professional autonomy, which up-or-out pressures can magnify into something close to an existential concern (Zhan and Chen, 2025).

### 6.3 The moderating role of organizational support

Organizational support moderated the association between algorithm anxiety and job burnout (H5 supported). The Johnson–Neyman results show that the positive anxiety–burnout association held at lower levels of support, weakened in the mid-range, and—above an organizational-support value of 5.36—reversed in sign. We read this reversal as suggestive rather than definitive. At a minimum it indicates that ample organizational support can offset the depleting association between anxiety and burnout; the stronger possibility, that anxiety becomes mobilizing under very high support, is theoretically plausible in light of the JD-R model, in which abundant resources can reframe demands as challenges (Bakker and Demerouti, 2017), but it rests on a cross-sectional pattern and needs replication before strong claims are warranted. Even read conservatively, the result deepens organizational support theory in the algorithmic-governance context (Eisenberger et al., 1986): organizational resources may not merely cushion technological stress but also reshape how teachers relate to algorithmic systems.

## 7 Theoretical contributions and practical implications

### 7.1 Theoretical contributions

This study makes several theoretical contributions. First, it brings perceived fairness of algorithmic decision-making, psychological safety, algorithm anxiety, and job burnout into a single explanatory framework, connecting two literatures—algorithmic governance and teachers’ occupational health—that have largely developed apart. Second, instead of treating fairness as a direct cause of burnout, it specifies and tests an internal pathway in which a cognitive resource (psychological safety) and an emotional response (algorithm anxiety) operate in sequence, so that fairness perceptions are linked to burnout through appraisal rather than through impact alone. This serial structure gives empirical content to the appraisal logic of the transactional theory of stress and shows that the two mediators are ordered rather than interchangeable. Third, the analysis identifies algorithm anxiety as the most influential mediator in this setting, which places teachers’ technology-related affect—not only their judgments of fairness—at the center of the burnout process and refines how the JD-R model applies to algorithmic demands. Fourth, by casting organizational support as a boundary condition and uncovering a support-dependent shift in the anxiety–burnout association, the study extends organizational support theory and the JD-R model into conditional territory, suggesting that the same technological stressor can carry different implications depending on the resources that surround it. These four contributions outline a technology–individual–organization account of occupational health in algorithmically governed universities.

### 7.2 Practical implications

The findings also speak to practice. The first priority is the fairness of algorithmic evaluation itself. Universities can treat distributive, procedural, and interactional fairness as explicit design requirements (Cropanzano and Ambrose, 2015), making decision rules transparent, explainable, and open to appeal, and embedding professional judgment in subjective tasks such as teaching assessment and promotion review so that measurable indicators do not displace qualitative contributions. An algorithm ethics committee that includes disciplinary experts, frontline teachers, and technical staff, together with periodic third-party fairness audits, can give teachers a real voice in how indicators are weighted and how results are explained.

Second, because psychological safety carries part of the association with burnout, fairness work should be paired with a climate that tolerates honest mistakes and supports development, so that teachers meet algorithmic uncertainty with engagement rather than defensive withdrawal. Third, given the central role of algorithm anxiety, universities are better served by treating such anxiety as a signal of system shortcomings than as individual technophobia. Routine, low-stakes monitoring (for example, anonymous surveys or focus groups), feedback channels that feed into algorithm revision, and support for teachers’ algorithmic literacy can all reduce the helplessness that fuels anxiety. Fourth, organizational support is best built as a layered ecosystem rather than a single program: institutional safeguards such as consultation and appeal channels, resource safeguards such as protected time and psychological assistance, and a culture of human–machine collaboration in which teachers see themselves as participants in, not merely subjects of, algorithmic governance. Because the buffering role of support varied across the sample, these measures can be differentiated—emphasizing explanation and participation for senior staff, and guidance and psychological support for early-career teachers—so that limited resources are directed where they most reduce burnout risk.

## 8 Limitations and conclusion

Several limitations qualify these conclusions and point to next steps. The design is cross-sectional, so the relationships reported here are associative, and the appraisal sequence implied by the model should be confirmed with longitudinal or experimental data. The measures were entirely self-reported, which leaves room for common method variance despite the diagnostic checks reported above; multi-source or multi-wave designs would strengthen inference. The sample was drawn from only two provinces, Shanghai and Gansu; although this contrast was chosen to widen variation, institutional type, disciplinary culture, and regional context may each shape how teachers perceive algorithmic fairness and experience burnout, so the findings should be generalized with care and re-examined across a broader range of institutions and regions. Finally, the sign reversal of the anxiety–burnout association at high organizational support, though intriguing, rests on cross-sectional evidence and awaits replication before it can bear strong theoretical weight. With these cautions in mind, the study constructed and tested a moderated chain-mediation model showing that perceived fairness of algorithmic decision-making is associated with lower job burnout among university teachers, largely through greater psychological safety and lower algorithm anxiety, and that organizational support conditions the final step from anxiety to burnout. It thereby offers an integrated, appraisal-based account of teachers’ occupational health in the algorithmic era, together with practical guidance for fairer and more supportive algorithmic governance in higher education.

## Data Availability

The raw data supporting the conclusions of this article will be made available by the authors, without undue reservation.
